# Supplementary material for: Content-rich biological network constructed by mining PubMed abstracts
Source: BMC Bioinformatics. 2004 Oct 8;5:147. doi: 10.1186/1471-2105-5-147 (PMC528731; doi:10.1186/1471-2105-5-147)
Supplement: Additional File 5 — The original Chilibot query results of the term "long-term potentiation (LTP)" and 22 other terms, limiting the latest references analyzed to the years 1990, 1995, 2000, and 2004. [file 1471-2105-5-147-S5.bz2 › chilibotAdditionalFile5/ltp2004/html/SYNAPSIN I_PI-3K.html]

 


 **SYNAPSIN I** and **PI-3K** 
  
Found 1 abstracts in PubMed,  **1 abstracts were retrieved and analyzed**.  


---

 Search Google  |
 PDF files only 
|  EDU domain only 

---

- J Biol Chem, 2003   ****Synapsin I** associated phosphatidylinositol 3 kinase mediates synaptic vesicle delivery to the readily releasable pool.** .
  Maintaining synaptic transmission requires replenishment of docked synaptic vesicles within the readily releasable pool RRP from synaptic vesicle clusters in the synapsin bound reserve pool.
  We show that synapsin forms a complex with phosphatidylinositol 3 kinase PI 3 kinase **[PI-3K]** in intact nerve terminals.
  synapsin associated kinase activity increases on depolarization.
  Disruption of either PI 3 kinase **[PI-3K]** activity or its interaction with synapsin inhibited replenishment of the RRP, but did not affect exocytosis from the RRP.
  Thus we conclude that a synapsin associated PI 3 kinase **[PI-3K]** activity plays a role in synaptic vesicle delivery to the RRP.
  This also suggests that PI 3 kinase **[PI-3K]** contributes to the maintenance of synaptic transmission during periods of high activity, indicating a possible role in synaptic plasticity.
